# Electrocardiographic Changes in Patients With Hypokalemia and Their Correlation With Serum Potassium Levels

**DOI:** 10.7759/cureus.91922

**Published:** 2025-09-09

**Authors:** Srinivasan Ramadurai, Visvarath Varadarajan, Shakthi Harini S, Thenappan T, Krithika V, Suresh Varadarajan

**Affiliations:** 1 General Medicine, Sri Ramachandra Institute of Higher Education and Research, Chennai, IND; 2 General Medicine, Government Stanley Medical College and Hospital, Chennai, IND; 3 Community Medicine, Sri Ramachandra Institute of Higher Education and Research, Chennai, IND

**Keywords:** ecg interpretation, ecg morphology, electrocardiography (ecg), hypokalemia, potassium

## Abstract

Background: Dyselectrolytemias can cause significant changes in cardiac electrophysiology detectable through electrocardiography (ECG). Potassium in particular has a strong effect on the cardiac membrane potential, leading to characteristic rhythm variations when potassium levels are reduced or elevated. However, the degree of correlation between specific ECG changes and the severity of hypokalemia remains variably described in the literature.

Objective: To study the association between the severity categories of hypokalemia and specific ECG waveform changes, and to examine the mechanisms by which hypokalemia may cause them.

Methods: During the study period of three months, ECGs from patients with varying levels of hypokalemia were obtained as per the inclusion and exclusion criteria after obtaining informed consent. ECGs were grouped according to corresponding potassium levels (mild 3-3.4 mEq/L, moderate 2.5-3 mEq/L, and severe <2.5 mEq/L). ECGs were analyzed for morphology and the durations of the waves, segments, and intervals. Statistical analysis was performed with a chi-square test to determine associations between individual ECG features and potassium levels.

Results: Univariate analysis showed no statistically significant relationship between the category of severity of hypokalemia and disordered heart rate (p = 0.292), the presence of a tall P wave (0.089), prolonged PR interval (0.310), and QRS width (0.075). There were statistically significant associations between the category of severity of hypokalemia and prolonged QT interval (p = 0.013), presence of ST segment depressions (p < 0.001), presence of T-wave flattening/inversions (p = 0.007), and presence of U waves (p < 0.001).

Conclusion: ECG changes, particularly the presence of U waves, ST depression, T-wave flattening/inversion, and QT prolongation, are reliable markers of hypokalemia severity, supporting the use of ECG as a rapid bedside diagnostic tool and guiding emergent management. The importance of identifying ECG changes and the mechanisms of these changes has been discussed.

## Introduction

Hypokalemia is defined as a serum potassium level below 3.5 mEq/L. It can further be divided into mild (3-3.4 mEq/L), moderate (2.5-2.9 mEq/L), and severe (<2.5 mEq/L) [1,2]. Common causes of hypokalemia include poor intake (such as starvation or anorexia), gastrointestinal losses (such as vomiting, diarrhea or continuous nasogastric drainage), renal losses (such as in renal tubular acidosis, diuretic use, hypomagnesemia or salt-wasting nephropathy), or internal redistribution (such as catecholamine excess, administration of insulin, alkalosis, hypokalemic periodic paralysis) [1].

Potassium plays an important role in the generation and propagation of the cardiac action potential. As such, alterations in the serum concentrations of potassium can lead to alterations in the transcellular potassium gradient, which can interfere with cardiac electrophysiology and precipitate life-threatening arrhythmias. In the case of hyperkalaemia, there may be bradyarrhythmias, sine wave morphology, ventricular fibrillation, pulseless electrical activity, or asystole; in hypokalemia, arrhythmias include conduction blocks, ventricular tachycardia, and torsades de pointes [2]. Electrocardiography (ECG) is a quick, cost-effective point-of-care modality that is still widely used to this day in the identification of cardiac electrophysiological disruptions. It is therefore important to be able to recognize the ECG changes associated with hypokalemia and to be able to understand the likely severity of the underlying electrolyte disorder, as this avoids delays in treatment that may occur when waiting for accurate laboratory measurement of serum potassium (such as due to hemolysed samples, sample transport time, laboratory processing time and time required to recheck a grossly abnormal value per protocol).

In this study, we aim to examine the various ECG changes associated with hypokalemia and the frequency with which they occur in different levels of hypokalemia.

## Materials and methods

This cross-sectional study was conducted over a period of three months (from March to May 2025) in the medical wards and ICUs at Sri Ramachandra Institute of Higher Education and Research in Chennai, India. The study was approved by the Institutional Ethics Committee (IEC-NI/21/APR/78/89). Consent was obtained for all study participants before the collection of data.

Inclusion criteria

All adult patients (aged 18 years or older) admitted to the medical wards or the ICU during the study period with serum potassium levels less than 3.5 mEq/L were reviewed. After informed consent was taken, the ECGs of consenting patients taken at the time of identification of hypokalemia were included in the study. Patients were also screened for multiple electrolyte disturbances (such as sodium, calcium, and magnesium) and for thyroid dysfunction.

Exclusion criteria

ECGs of pediatric patients (age less than 18 years) and patients with known ECG abnormalities due to intrinsic cardiac disease or medication use, as well as patients with thyroid disorders or electrolyte abnormalities other than hypokalemia, were excluded from this study. This exclusion criterion was observed to ensure that any observed ECG changes could be attributable to hypokalemia alone, and not to any other electrolyte or metabolic disorder typically concomitant with hypokalemia (such as hypomagnesemia). ECGs with excessive artefact, poor quality leading to unreadability, or other mechanistic and logistic issues were also excluded from the study.

Statistical analysis

A total of 100 patients were recruited this way. Patients were then placed into one of three groups based on their serum potassium levels at admission: those with mild hypokalemia (3.0-3.4 mEq/L) were assigned to group A; those with moderate hypokalemia (2.5-2.9 mEq/L) to group B; and those with severe hypokalemia (<2.5 mEq/L) to group C. Characteristics of the morphology of ECG waves, segments, and intervals were compared to the hypokalemia severity of each patient. Specific characteristics (with the values considered normal) measured include heart rate (60-100 bpm), P wave height (<2.5 mm) and width (<120 ms), PR interval (≤200 ms), QRS width (≤110 ms), QRS morphology, corrected QT (QTc) interval (≤440 ms in males, ≤460 ms in females), presence of ST segment depression ≥1 mm, presence of T-wave flattening or inversions and presence of U waves. In the context of the presence of U waves, especially when the U wave was fused with the T wave and a distinct QT interval could not be made out, the QU interval was measured instead, as per previous studies showing that the QU interval is consistent with action potential duration [3].

Descriptive statistical analysis was done using the mean and standard deviation for heart rate as a continuous variable, the median and interquartile range for potassium levels, and percentages for qualitative data. Inferential statistics was done with univariate analysis using the chi-square test. As three groups of hypokalemia severity were factored into the analysis, the derived chi-square value was compared to the chi-square distribution table for two degrees of freedom, and p-values were obtained accordingly. These p-values were compared to an α of 0.05, and thus a p-value of <0.05 was considered to be statistically significant. The presence of the parameters for which the p-values were statistically significant was considered to be correlated with the severity of hypokalemia. Thus, clinically, the presence of these electrocardiographic dysmorphologies may be reasoned to be a potential marker of the degree of hypokalemia. Microsoft Excel (Microsoft® Corp., Redmond, WA, USA) was used for recording data, and analysis was done using the IBM SPSS Statistics for Windows, Version 25 (Released 2021; IBM Corp., Armonk, New York, United States).

## Results

A total of 100 ECGs satisfying all inclusion criteria and not satisfying any exclusion criteria were analyzed. The results are presented in Table 1.

**Table 1:** Comparison of potassium levels and ECG features across categories of hypokalemia severity. The bold values represent significant p-values (p < 0.05). P-values calculated using the chi-square test. IQR: interquartile range; SD: standard deviation; mEq/L: milliequivalent per liter; bpm: beats per minute; ms: milliseconds

Characteristic	Group A (n = 41)	Group B (n = 39)	Group C (n = 20)	P-value
Gender ratio, male:female (%)	23:18 (56.09)	21:18 (53.84)	11:9 (55)	-
Potassium, mEq/L: median (IQR)	3.2 (0.2)	2.7 (0.3)	2.2 (0.325)	-
Heart rate, bpm: mean (SD)	91.8 (18.8)	98.1 (25.8)	98.4 (24.8)	0.36
Heart rate (<60 bpm): number (%)	0 (0)	1 (2.6)	1 (5)	0.292 (for heart rate category)
Heart rate (60-100 bpm): number (%)	30 (73.2)	22 (56.4)	10 (50)	-
Heart rate (>100 bpm): number (%)	11 (26.8)	16 (41)	9 (45)	-
Tall P waves (≥2.5 mm): number (%)	0 (0)	3 (7.7)	0 (0)	0.089
Broad P waves (≥120 ms): number (%)	1 (2.4)	1 (2.6)	1 (5)	0.842
Prolonged PR interval (>200 ms): number (%)	1 (2.4)	1 (2.6)	2 (5)	0.310
QRS width (>110 ms): number (%)	1 (2.4)	4 (10.3)	4 (20)	0.075
QTc interval (≥440 ms in males, ≥460 ms in females): number (%)	17 (41.5)	24 (61.5)	16 (80)	0.013
ST-segment depression: number (%)	4 (9.75)	15 (38.5)	15 (75)	<0.001
T-wave flattening/inversion: number (%)	8 (20)	15 (39)	12 (60)	0.007
Presence of U wave: number (%)	3 (7.3)	14 (35.9)	16 (80)	<0.001

Heart rate was analyzed both as a continuous variable and as a categorical variable consisting of heart rate categories (bradycardia, normal heart rate, and tachycardia). A total of 36 ECGs across all groups had tachycardia, but a significant correlation with the severity of hypokalemia was not noted for the continuous heart rate (p = 0.36) or when analyzed qualitatively (p = 0.292). There was also no statistically significant association between hypokalemia severity and the presence of tall P waves (p = 0.089), prolonged PR interval (p = 0.310), or QRS widening (p = 0.075).

QTc intervals calculated using Bazett's formula had better correlation with hypokalemia severity (p = 0.013); as potassium decreases, QTc appears to increase in inverse proportion. T-wave abnormalities, including flattening and inversion, were also correlated with the severity of hypokalemia (p = 0.007). A total of 60% of group C patients had inversions compared to 39% in group B and 20% in group A. All eight patients in group A had T-wave flattening without frank inversions.

Almost twice as many group C patients with ECGs showed ST depression percent-wise compared to group B, which in turn had almost four times as many patients whose ECGs showed ST depression as group A in terms of percentage (p < 0.001). U waves appeared in 80% of group C patients’ ECGs (p < 0.001).

**Figure 1:**
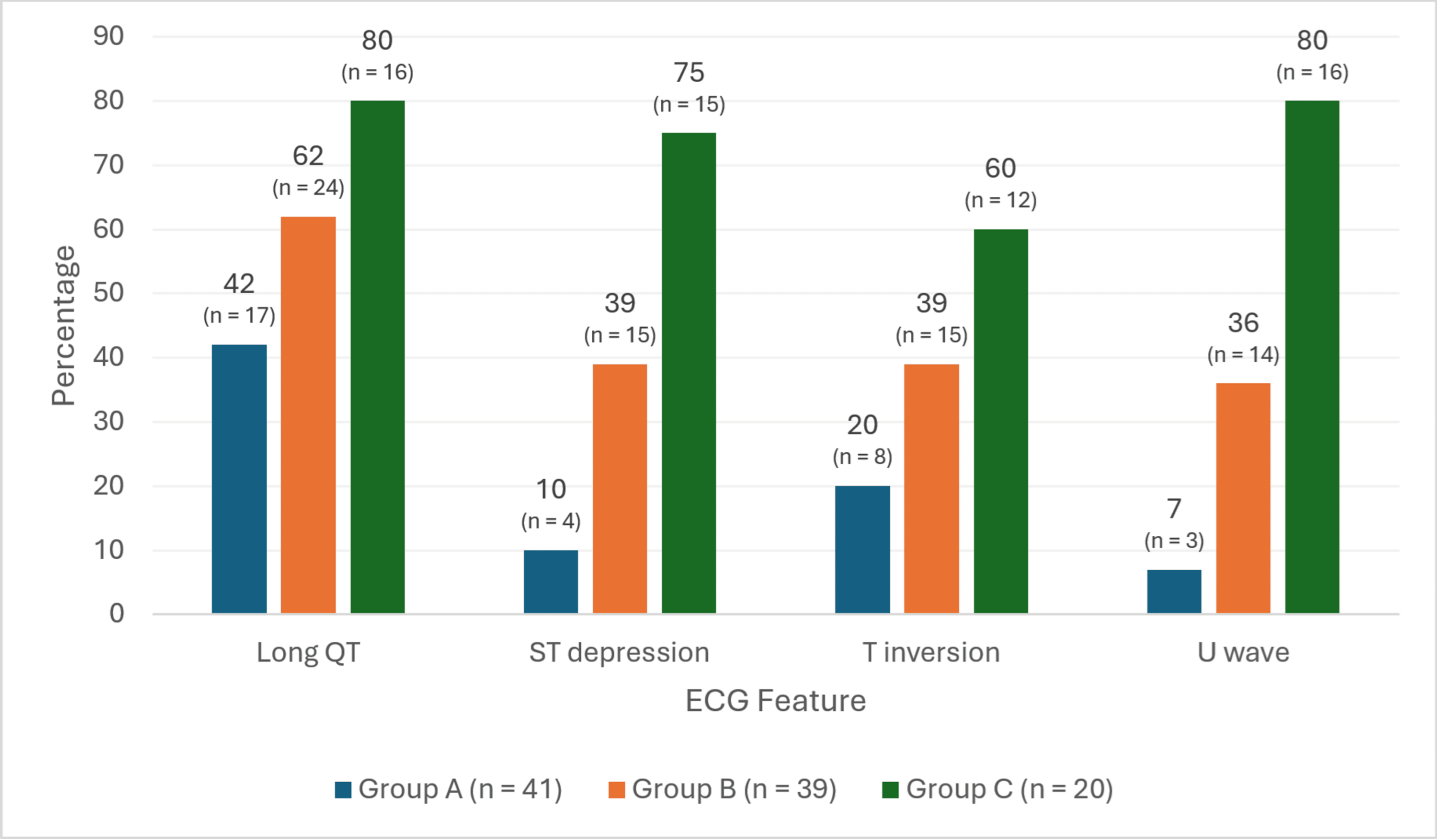
Comparison of frequencies of statistically significant ECG dysmorphologies across groups.

Figure 1 shows the increasing frequency of QT prolongation, ST depression, T-wave changes, and U waves with worsening hypokalemia severity.

## Discussion

In group A, among patients with mild hypokalemia, the predominant findings were prolonged QTc interval (41%) and T-wave flattening/inversion (20%). In group B, patients with moderate hypokalemia most commonly had prolonged QTc (61.5%), ST segment depression (38.5%), T-wave changes (39%), and U waves (35.9%). The patients in group C with severe hypokalemia also had high rates of prolonged QTc (80%), ST segment depression (70%), T-wave inversion (60%), and U waves (80%).

Potassium is the most important intracellular cation and plays a great role in maintaining resting membrane potential (RMP), especially in opening delayed rectifier channels. Hypokalemia leads to a more negative RMP. As a result, during electrical diastole, the gap between the threshold potential (TP) and RMP increases, resulting in decreased membrane excitability. Due to low extracellular potassium levels, fewer outward rectifier current channels are recruited, causing a more gradual slope of phase 2 of the cardiac action potential and the lower velocity of phase 3 [4]. This manifests as an increase in the action potential duration and a delay in repolarization. This long action potential duration leads to a decrease in the difference between the TP and RMP during the terminal phase of the action potential and an increase in the relative refractory period. The increase in relative refractory period is associated with ectopy and genesis of arrhythmias.

Effect of hypokalemia on various channels

There are three major potassium channels involved in cardiac action potential. They are the inward rectifier potassium channel (I_K1_), delayed rectifier-like potassium channel (I_Kr_), and transient outward potassium current (I_to_) [4]. In hypokalemia, delayed rectifier and transient outward potassium current channels are rapidly inactivated. There is also internalization and degradation of delayed rectifier channels within hours of the onset of hypokalemia [5-7].

The rate of sodium-potassium-ATPase pump in transporting ions depends upon the saturation of the binding sites of corresponding ions. When the extracellular concentration of potassium falls, the saturation of binding sites by potassium ions on the sodium-potassium pump also falls, leading to a decrease in the functional capacity of the pump. This leads to increased intracellular sodium concentration.

Intracellular calcium overload and calcium/calmodulin-dependent protein kinase (CaMK) activation

Decreased efflux of potassium leads to increased influx of calcium through the calcium channels. The passive sodium-calcium channel is compromised by the high intracellular sodium concentration secondary to sodium-potassium pump inhibition, leading to a further increase in intracellular calcium concentration. This, in turn, causes activation of CaMK, which phosphorylates sodium, L-type, and ryanodine receptor calcium channels. This leads to the prolongation of repolarization and lengthening of the effective refractory period, leading to the generation of early after-depolarizations (EAD) and delayed after-depolarizations (DAD), causing increased automaticity and arrhythmogenicity [8-10].

ECG manifestations of hypokalemia

The earliest sign of hypokalemia is a change in T-wave morphology. Due to delayed and decreased rectifier current potential, there is differential action potential prolongation of different layers of the Purkinje system. This leads to a change in voltage gradients and causes variable T-wave morphologies such as flattened, inverted, biphasic, and triphasic patterns. Often, the T wave initially decreases in height, then becomes isoelectric and later inverts [11].

U waves are an important sign of hypokalemia. These are positive deflections following the T wave, best seen in the precordial leads (especially V2-V4). The most accepted theory regarding the genesis of the U wave describes that the action potential in Purkinje fibers is relatively longer than that in the ventricular myocytes, resulting in dispersion of repolarization and the U wave [12,13]. The QTc interval represents the sum of ventricular depolarization and repolarization and is usually prolonged in moderate to severe hypokalemia [14,15]. However, what is measured as a prolonged QTc interval may in fact be a QU interval (the interval from the beginning of the QRS complex to the end of the U wave), as the U wave may be fused into the T wave, leading to a wider-appearing T wave. All the same, the duration of the QU wave is a risk for torsades de pointes (28% of cases thereof being associated with hypokalemia), as the fused T-U wave still represents a delay in repolarisation and predisposes the affected heart to the R-on-T phenomenon, where a ventricular depolarisation potential initiated during the latter half of the repolarisation period can lead to ventricular ectopic beat generation and ventricular tachydysrhythmia [16-20]. Evaluating for hypokalemia and timely potassium repletion are therefore an important part of the management of these arrhythmias.

ST-segment depression is frequently seen, which might be mistaken for ischemia. This results from the prolonged repolarization phase of the cardiac action potential [21]. In severe hypokalemia, the P wave may also become prominent, and the PR interval may be prolonged due to delayed atrial and atrioventricular (AV) nodal conduction [22].

**Figure 2:**
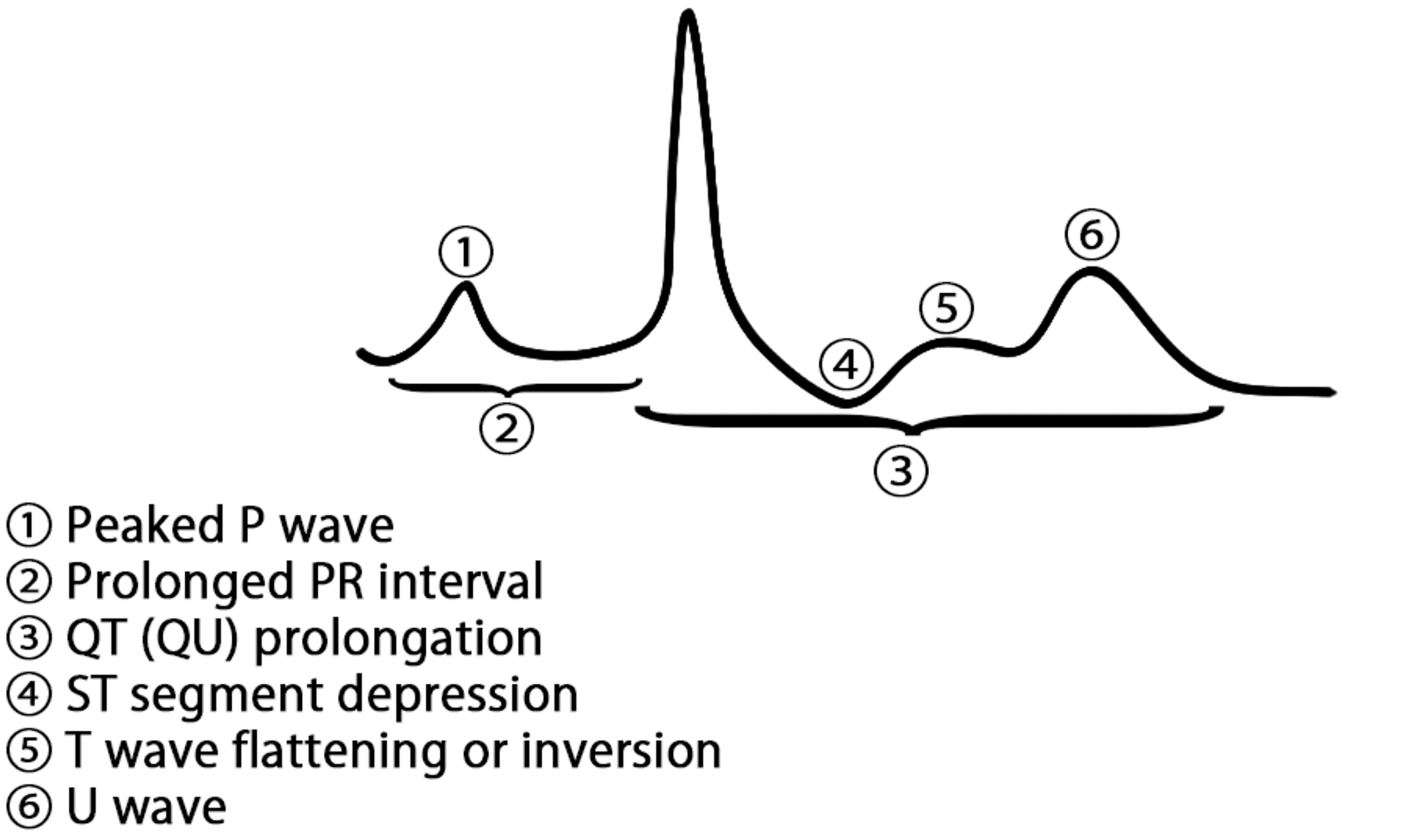
ECG signs of hypokalemia. Illustrative graphic manually drawn with text added using Photoshop (Adobe Inc., San Jose, California, USA). Image credit: Dr. Srinivasan Ramadurai

**Figure 3:**
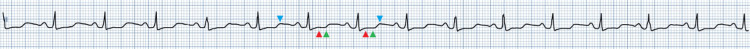
ECG rhythm strip showing ST depression (red arrowheads), T-wave flattening (green arrowheads), and U waves (blue arrowheads). Patient ECG digitized using the PMCardio app (Powerful Medical, Bratislava, Slovakia). Image credit: Dr. Srinivasan Ramadurai

**Figure 4:**
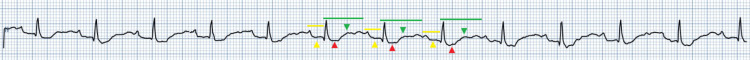
ECG rhythm strip showing PR prolongation (yellow arrowhead, with yellow bar showing duration), ST depression (red arrowhead), and QT/QU prolongation (green arrowhead, with green bar showing duration). Patient ECG digitized using the PMCardio app (Powerful Medical, Bratislava, Slovakia). Image credit: Dr. Srinivasan Ramadurai

**Figure 5:**
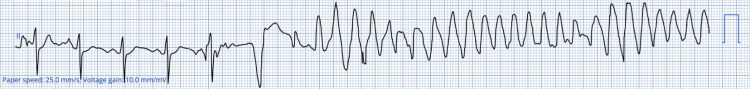
ECG rhythm strip showing a rhythm with features suggestive of hypokalemia devolving into polymorphic ventricular tachycardia. Patient ECG digitized using the PMCardio app (Powerful Medical, Bratislava, Slovakia). Image credit: Dr. Srinivasan Ramadurai

A pictorial representation of the ECG signs of hypokalemia is in Figure 2. Examples of ECGs with various signs of hypokalemia are also given in Figures 3-5.

Study limitations

The major limitation of this study is the sample size. Features such as P wave height and QRS width may also share a statistically significant difference across hypokalemia severity groups, which may be uncovered when studied with a larger sample size. For a target power of 90%, the study was adequately powered for an α = 0.05 and two degrees of freedom for the ECG findings of ST depression (>99%), T-wave flattening (82%) and U wave presence (>99%), with a borderline power for QT prolongation (75%); however, the study is underpowered for the remaining parameters, with an ideal sample size of 411 to reach adequate power for prolonged PR in particular, using Cohen's formula. Larger-scale, multicenter studies over a longer period of time that aim to determine the ability of ECG morphology to predict serum potassium values may be able to predict the risk of hyperkalemia, given a set of ECG abnormalities with better precision and in a manner that can be more generalizable to the population at large. Additionally, the cross-sectional nature of the study does not allow for the establishment of a direct causal link between hypokalemia and the ECG findings studied. Traditional experimental methods may be infeasible in humans. However, a study constructed with the data of patient who were known to be normokalemic with normal ECGs and who subsequently developed hypokalemia (due to their disease condition or treatment) with new onset ECG changes could provide particularly valuable information in this regard.

## Conclusions

Intracellular and extracellular ions play a significant role in cardiac action potential. Potassium is the most abundant cation in the body and is predominantly in the intracellular compartment. Potassium plays a major role in maintaining the RMP, initiation of depolarization, and repolarization. Changes in the extracellular concentration of potassium have profound effects on the cardiac cycle, leading to conduction abnormalities and fatal arrhythmias. The findings of this study underscore the value of the ECG as a rapid diagnostic tool for hypokalemia, especially in the context of assessment for potassium replacement therapy in patients with severe rhythm changes in low-resource or acute care settings. Therefore, it is prudent for all physicians to be familiar with associated ECG changes for timely diagnosis and to guide therapeutic interventions.
